# Modeling of *Anopheles minimus* Mosquito NADPH-Cytochrome P450 Oxidoreductase (CYPOR) and Mutagenesis Analysis

**DOI:** 10.3390/ijms14011788

**Published:** 2013-01-16

**Authors:** Songklod Sarapusit, Panida Lertkiatmongkol, Panida Duangkaew, Pornpimol Rongnoparut

**Affiliations:** 1 Department of Biochemistry, Faculty of Science, Burapha University, Chonburi 20131, Thailand; 2 Department of Biochemistry, Faculty of Science, Mahidol University, Bangkok 10400, Thailand; E-Mails: prunuspersica@gmail.com (P.L.); pornpimol.ron@mahidol.ac.th (P.R.); 3 Faculty of Animal Sciences and Agricultural Technology, Silpakorn University, Petchaburi IT Campus, Petchaburi 76120, Thailand; E-Mail: panida.d@su.ac.th

**Keywords:** *Anopheles minimus* mosquito, cytochrome P450 oxidoreductase (CYPOR), structure, FAD and NAD(P)H bindings

## Abstract

Malaria is one of the most dangerous mosquito-borne diseases in many tropical countries, including Thailand. Studies in a deltamethrin resistant strain of *Anopheles minimus* mosquito, suggest cytochrome P450 enzymes contribute to the detoxification of pyrethroid insecticides. Purified *A. minimus* CYPOR enzyme (AnCYPOR), which is the redox partner of cytochrome P450s, loses flavin-adenosine di-nucleotide (FAD) and FLAVIN mono-nucleotide (FMN) cofactors that affect its enzyme activity. Replacement of leucine residues at positions 86 and 219 with phenylalanines in FMN binding domain increases FMN binding, enzyme stability, and cytochrome *c* reduction activity. Membrane-Bound L86F/L219F-AnCYPOR increases *A. minimus* P450-mediated pyrethroid metabolism *in vitro.* In this study, we constructed a comparative model structure of AnCYPOR using a rat CYPOR structure as a template. Overall model structure is similar to rat CYPOR, with some prominent differences. Based on primary sequence and structural analysis of rat and *A. minimus* CYPOR, C427R, W678A, and W678H mutations were generated together with L86F/L219F resulting in three soluble Δ55 triple mutants. The C427R triple AnCYPOR mutant retained a higher amount of FAD binding and increased cytochrome *c* reduction activity compared to wild-type and L86F/L219F-Δ55AnCYPOR double mutant. However W678A and W678H mutations did not increase FAD and NAD(P)H bindings. The L86F/L219F double and C427R triple membrane-bound AnCYPOR mutants supported benzyloxyresorufin *O*-deakylation (BROD) mediated by mosquito CYP6AA3 with a two-to three-fold increase in efficiency over wild-type AnCYPOR. The use of rat CYPOR in place of AnCYPOR most efficiently supported CYP6AA3-mediated BROD compared to all AnCYPORs.

## 1. Introduction

The reemergence of malaria, a disease transmitted to humans by mosquitoes, is associated with vector resistance in many tropical countries due to the persistent use of pyrethroid insecticides [1,2]. The insecticide resistance has been reported in *Anopheles minimus* mosquito, one of the malaria primary vectors in Thailand [3]. A common mechanism for insecticide resistance involves an elevation in insecticide detoxification. Cytochrome P450 monooxygenases (P450) are important enzymes in pyrethroid metabolism and have been implicated in insecticide resistance in many insects [4]. In *A. minimus*, an elevated level of CYP6AA3 and CYP6P7 transcripts has been detected during selection for deltamethrin resistance [5,6]. Heterologously expressed CYP6AA3 and CYP6P7 enzymes in an insect-baculovirus system have shown capability to specifically metabolize type I and type II pyrethroids *in vitro* [7–9].

Catalysis by P450 enzymes requires an electron supplement from NADPH-cytochrome P450 oxidoreductase enzyme (CYPOR), a membrane-bound enzyme that transfers electrons, from NADPH to P450s via FAD and FMN cofactors [10–12]. RNA interference indicated the influential role of mosquito CYPOR enzyme in permethrin resistance in *A. gambiae*, a major malaria vector in Africa [13]. Thus, studies of CYPOR properties in *A. minimus*, may provide important information for the malarial vector control program in Thailand.

The membrane-bound *A. minimus* CYPOR (flAnCYPOR) cDNA has been isolated and expressed in *Escherichia coli* [7]. The purified flAnCYPOR enzyme can support baculovirus-expressed CYP6AA3 and CYP6P7 to metabolize pyrethroid insecticides and benzyloxyresorufin fluorescent substrate *in vitro*, but the purified enzyme is prone to lose FAD and FMN cofactors compared to rat CYPOR [7–9,14,15]. In addition, higher trypsin sensitivity of AnCYPOR indicates a more open conformation or high flexibility of the soluble Δ55AnCYPOR enzyme compared to rat CYPOR structure. Unlike rat and house fly CYPORs, the open/highly flexible structure of AnCYPOR could explain that AnCYPOR loosely binds flavin cofactors and can be reconstituted with exogenous FMN and FAD *in vitro* [14–17]. Substitutions of leucine 86 and leucine 219 with phenylalanine in the FMN-binding domain generate a L86F/L219F double mutant of both soluble (Δ55) and membrane-bound (fl) forms that improve FMN retention, but remain loosely bound to FAD cofactor. Binding of FAD is not improved when phenylalanine at 456 is replaced with the conserved alanine residue in the FAD-binding domain of the enzyme [14,15]. The L86F/L219F mutation increases enzyme stability and turnover number without drastically changing kinetic mechanism and substrate-binding constants, NADPH *K*_m_, and cytochrome *c K*_m_ [14,15]. Consequently, a higher catalytic efficiency of L86F/L219F-flAnCYPOR increases CYP6AA3-mediated deltamethrin degradation activity *in vitro* [15]. Addition of exogenous FAD contributes to a greater increase in CYP6AA3-mediated activity supported by the wild-type and L86F/L219F mutant flAnCYPOR enzymes. In this context, why the *A. minimus* CYPOR is enormously different from rat CYPOR in stability, activity in its native form and in the ease in which flavin cofactors are lost remains unanswered.

Recently, investigation of *A. gambiae* CYPOR, AgCYPOR, which shares 93% of its amino acid sequence with *A. minimus* CYPOR, has indicated a loss of both FMN and FAD cofactors in the purified AgCYPOR enzyme. Moreover, AgCYPOR binds NAD(P)H differently from human CYPOR [18], suggesting substantially different enzymatic properties in mosquito CYPORs. Functional analyses of mosquito and mammalian CYPOR in combination with a structural comparison may help to explain different properties. In this study, comparative modeling of AnCYPOR was performed and the predicted models were compared to known crystal structure of rat CYPOR [10]. Based on sequence analysis and model comparison, two residues that might affect FAD binding and enzyme catalysis, C427 and W678, were selected to generate triple mutants, in addition to L86F and L219F. Biochemical properties of L86F/L219F/C427R, L86F/L219F/W678A, and L86F/L219F/W678H soluble Δ55-triple mutants and catalysis by L86F/L219F double and C427R triple membrane-bound mutants in support of BROD mediated by CYP6AA3 were investigated. Moreover, as a primary step to understand different properties of AnCYPOR and rat CYPOR in electron transfer to P450 partner enzyme, we employed rat CYPOR in place of mosquito CYPOR as a redox partner enzyme of mosquito P450 in the reconstitution enzymatic assay. Our results have contributed to an understanding of the typical nature of mosquito CYPOR compared to rat CYPOR, and its efficacy in electron transfer in mosquito P450-mediated metabolisms.

## 2. Results and Discussion

### 2.1. Overall Structure of AnCYPOR

**Figure 1:**
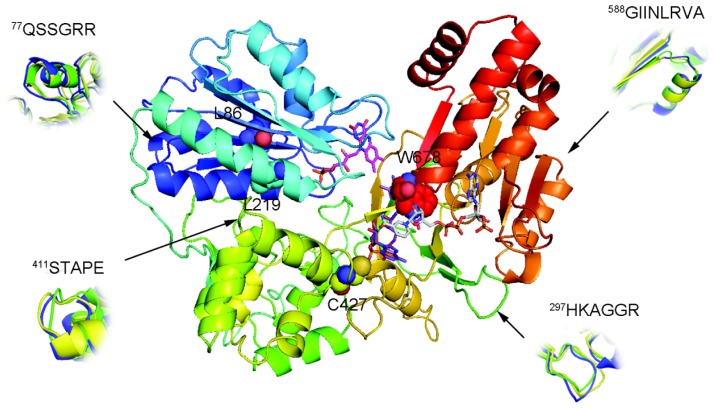
Homology model of wild-type *Anopheles minimus* CYPOR (AnCYPOR). Wild-Type AnCYPOR model demonstrates conserved regions of the flavin mono-nucleotide (FMN)-binding domain, the connecting domain, and the flavin-adenosine di-nucleotide (FAD)/NAD(P)H-binding domain that are colored blue, green, and red, respectively. The mutated positions are labeled and shown in spheres. Arrows indicate deviations among the structures of template rat CYPOR (PDB:1JA1) (green), wild-type AnCYPOR (blue), and double mutant AnCYPOR (yellow) in the FMN-binding domain, the connecting domain, and the FAD/NAD(P)H-binding domain. The cofactor FMN, FAD, and NADPH are represented by magenta, purple, and grey sticks, respectively.

A predicted AnCYPOR homology model generated using rat CYPOR as a template (pdb code: 1JA1, [19]) was chosen based on a consensus judgment of discrete optimized protein energy (DOPE) and residue specific all-atom probability discriminatory function (RAPDF) scores. Figure 1 shows an oval, bowl-like 3D structure of the AnCYPOR homolog in the presence of NADP^+^, FAD, and FMN. The overall AnCYPOR structure contains three structural domains including FMN-binding, FAD/NADP^+^-binding, and connecting domains. The FMN, FAD and NADP(H) are positions in the middle of the structure as found in rat CYPOR [10,19]. The model has an overall ProSA-z score of −11.01. Ramachandran plot analysis revealed 88.7% and 0.6% of residues in most favorable and disallowed regions, respectively (Figure S1). Three residues (V256, N503, and E506) residing in disallowed region locate in the connecting domain which functions to bring the two flavins together and modulate electron transfer between them [10,19,20]. The crystal structure of rat CYPOR lacks 63 *N*-terminal amino acids and starts with Val64, so the corresponding first residue in AnCYPOR is Thr64. Although structure of the membrane-binding region of AnCYPOR could not be determined in the model, sequence alignment indicated a clear difference in membrane-binding sequence from rat CYPOR (Figure S2).

Superimposition of the wildtype- and L86F/L219F-AnCYPOR with rat CYPOR showed topology differences in all domains (Figure 1). Deviations are found in one location in FMN-binding domain (^77^QSSGRR, the connecting loop between helix A and β-sheet 1), one in FAD-binding region (^297^HKAGGR at the tip of β-sheet 8), one loop in NAD(P)H binding region (^588^GIINLRVA), and ^411^STAPE at the tip of helix K in connecting domain. These differences may reflect enzyme properties and conformational change during AnCYPOR catalysis compared to rat CYPOR.

### 2.2. Predicted FAD-Binding Region

**Figure 2:**
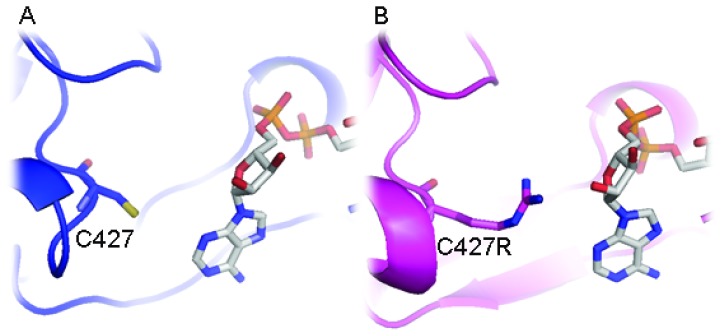
Binding interaction with FAD in AnCYPOR. Replacement of cysteine (**A**) with arginine (**B**) could result in more molecular interactions between the enzyme and FAD in mutant than in wild-type AnCYPOR in correspond to interactions towards FAD previously described in rat CYPOR [10]. Wild-Type and C427R triple mutant AnCYPOR are shown in blue and magenta cartoons, respectively. FAD is represented by grey stick. Nitrogen, oxygen, phosphorus, and sulfur are colored blue, red, orange, and yellow, respectively.

**Figure 3:**
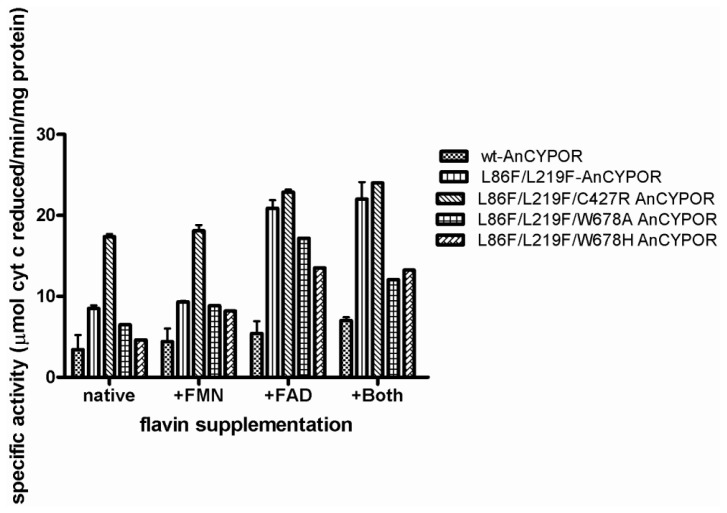
Specific activity of Δ55AnCYPOR enzymes with cytochrome *c* substrate. Specific activity is expressed as μmol of substrate reduced/min/mg of protein. Data are the average of duplicate measurements. Protein concentration was determined using Bio-Rad protein assay reagent and bovine serum albumin (BSA) as standard. When exogenous cofactors were tested, 2 μM of each of exogenous flavin cofactors were added and pre-incubated with enzyme in each assay reaction.

Although replacement of two leucine residues successfully increases FMN binding, possibly through altered topology of the loop at ^77^QSSGRR that superimposes well with rat structure than wild-type AnCYPOR, the L86F/L219F-Δ55AnCYPOR remains loosely bound to FAD cofactor. Therefore, a topology change in ^297^HKAGGR of L86F/L219F from wild-type AnCYPOR to that of rat CYPOR may not affect FAD binding (Figure 1). Relative to that of rat CYPOR structure, the re-side and the si-face of FAD ring are similarly stacked by the indole ring of W678 and the si-face by Y459 in AnCYPOR. The interaction of FAD isoalloxazine ring with side chains of S460, T475, A476 and V474 of AnCYPOR is also similar to rat CYPOR [10]. The rest of the FAD molecule lies at the interface between the FAD-binding domain, but polypeptide chains surrounding FAD show variable degrees of homology with rat CYPOR (Figure 1 and supplemental Figure 2). In addition, residues participating in stabilization of FAD binding in AnCYPOR are notably different from rat CYPOR. Residues observed in rat CYPOR are R424, R454, T491, Y478 and V489 [10], while in AnCYPOR they are R457, T494, Y481 and V492. The rat R424, which is important for interaction with FAD, is missing from AnCYPOR. Instead, a Cys residue was observed at position 427 (Figure S2). The short chain sulfhydryl-group of C427 might drastically abolish interaction with FAD, leading to a loose binding of FAD to the enzyme (Figure 2). Recently, disruption of local H bonding with FAD pyrophosphate moiety leading to weaker FAD binding, unstable protein, and loss of catalytic activity has been reported in V492E and R457H two naturally occurring missense mutations of human CYPOR [21]. Thus we replaced Cys with Arg together with L86F/L219F and generated a soluble triple mutant (L86F/L219F/C427R-Δ55AnCYPOR). The triple mutant could increase FAD binding about 1.5 folds of double mutant L86F/L219F-Δ55AnCYPOR, and about 1.3 folds compared to the wild-type-Δ55AnCYPOR (Table 1). The NADPH-dependent cytochrome *c* reduction activity in C427R triple mutant without supplementation of FAD was about two and four folds higher than L86F/L219F double mutant and wild-type-Δ55AnCYPORs, respectively (Figure 3). Supplementation with FAD reduced the difference in the cytochrome *c* reduction activity between the C427R triple mutant and the double mutant. The activity of this triple mutant enzyme is about 0.5 fold of rat CYPOR activity (51.5 μmol/min/mg) [17]. The results suggest a role of C427 in FAD binding and as a consequence increased AnCYPOR catalysis. Supplementation of FAD could further elevate the activity of C427R triple soluble mutant, indicating another factor might also influence FAD binding. The absence of cytochrome *c* reduction activity in the reaction omitted AnCYPOR enzyme excludes the possibility of an external electron transfer pathway to cytochrome *c* by exogenous flavins (unreported data). This increase in enzymatic activity of mutant AnCYPORs upon FAD supplement during enzyme catalysis had been reported in Y459A and V492E human mutant CYPOR enzymes that are loosely bound to FAD [22,23]. In addition, the C427R triple mutant had similar *K*_m_ values for NADPH and cytochrome c substrates to those of L86F/L219F double mutant, but its catalytic electron transfer rate (*V*_max_) was approximately two folds higher (data not shown). Nonetheless *K*_m_ values of L86F/L219F double and C427R triple mutants were not substantially different from wild-type-Δ55AnCYPOR indicating that the mutations did not significantly alter structure or substrate binding mode of enzyme (Table 2). The *K*_m_ values for cytochrome *c* and NADPH of wild-type-Δ55AnCYPOR in this study is noticeably lower than that previously reported [14] due to enzymatic assays performed in this study were in low ionic strength (0.1 M Tris pH 7.5) condition, while in previous report the assays were at high ionic strength (0.3 M Potassium phosphate pH 7.7). Effect of ionic strength on substrate binding of CYPOR has also been reported [11].

### 2.3. FAD/NADPH Binding Residues in AnCYPOR

Unlike FMN- and FAD-binding regions, there is no substantial alteration among residues in their roles in NADPH binding (R301, S597, R598, K603, Y605, T607, N636 and M637) and catalysis (S459, C631 and D676) in AnCYPOR model structure. However, one minor difference in rotameric conformation from rat CYPOR structure was found in NAD(P)H binding domain at the end of β-sheet 21 (^678^WS), located at the nicotinamide-binding site and covering the isoalloxazine ring of FAD cofactor. This conserved tryptophan (Trp) residue has been proposed to flip and facilitate nicotinamide binding and thus hydride transfer [10,19]. To investigate the contribution of such difference in rotameric conformation in nicotinamide binding and catalysis of AnCYPOR, we replaced W678 with alanine and histidine as both replacements play critical role in catalytic activity and nicotinamide binding of human and rat CYPORs [24,25].

In contrast to C427R construct, the Ala and His replacements at W678, (L86F/L219F/W678A-Δ55AnCYPOR and L86F/L219F/W678H-Δ55AnCYPOR) neither increased FAD binding nor cytochrome *c* reduction activity (Table 1 and Figure 3). However, cytochrome *c* reduction activity was decreased compared to L86F/L219F double mutant and C427R triple mutant. Mutations with aliphatic (W678A) and planar (W678H) residue substitutions decreased catalytic activity to 77% and 55% of that of L86F/L219F double mutant, respectively. The decrease in cytochrome *c* activity has been reported in the W676A and W676H human CYPOR mutants that retain 5 and 22% of cytochrome *c* activity, respectively [24,25]. Moreover, non-linear regression steady-state kinetic analysis using cytochrome *c* as an electron acceptor in this study revealed that removal of bulky Trp residue in W678A and W678H increased NADPH binding affinity by two- to three-fold compared to wild-type and L86F/L219F mutant AnCYPOR enzymes, but not that to NADH (Table 2). This differs from human CYPOR mutants in which both W676A and W676H mutants lower both NADPH *K*_m_, and NADH *K*_m_ [24,25]. Specific charge interactions of amino acid residues with 2′-phosphate of NADPH are responsible for discrimination against binding of NADH [24]. Thus, it is conceivable that W678 is only crucial for catalysis and NADPH binding in AnCYPOR, and not nicotinamde selectivity of NAD(P)H and NADH substrates. Whether deviation we observed at ^588^GIINLRVA (Figure 1) might involve in 2′-phosphate binding of NADPH is not known. This deviation might explain low binding affinity of *A. minimus* CYPOR to 2′,5′-ADP column compared to human and rat CYPOR [18,26]. Values of 2′AMP *K*_i_ for AnCYPOR are four-fold higher than those for rat CYPOR, suggesting significant differences in NAD(P)H binding property between rat CYPOR and AnCYPOR [14,15]. Low binding affinity to 2′,5′ADP column has also been observed for the mosquito AgCYPOR [18]. A two-fold higher IC_50_ in suppression of AgCYPOR by 2′AMP has also been reported compared to human CYPOR [18]. It is thus speculated that W678 could affect electron transfer rate and electron transfer activity, while dramatic change in polypeptide chain that specifically interact with NAD(P)H such as that of ^588^GIINLRVA might result in low nucleotide (2′,5′-ADP, 2′-AMP and NAD(P)H) binding affinity. However, the amino acids in the ^588^GIINLRVA polypeptide chain are distant from those of rat CYPOR.

Further mutational investigation of this polypeptide on nicotinamide selectivity may provide important information that could help to understand the catalytic basis of AnCYPOR.

### 2.4. Electron Transfer Step Is a Rate-Limiting Step in Mosquito P450 Metabolism

As P450 catalysis requires electron supplement from CYPOR, therefore, such a mutation that affects cytochrome *c* reduction activity could affect the P450-mediated oxygenase reaction *in vitro* [27]. This is supported by an increased efficiency of L86F/L219F-flAnCYPOR and L86F/L219F/C427R-flAnCYPOR mutants in supporting BROD mediated by mosquito CYP6AA3 enzyme by 2.4 and 3 times, respectively compared to wild-type (Table 3). Moreover rat CYPOR which has higher cytochrome *c* reduction activity than wild-type AnCYPOR and all AnCYPOR mutants could increase mosquito P450 activity more than wild-type flAnCYPOR by 5.5 times. These increases in velocity by L86F/L219F, C427R mutants, and rat CYPOR had no effect on substrate binding value (*K*_m_). The results suggest that *in vitro* mosquito P450 activity is an electron transfer rate dependent reaction. In human CYPOR, the loss of FAD and FMN from its binding site caused by mutation is the major cause of diminished P450 activity. For example, the R457H and V492E mutations at the FAD-binding site in human CYPOR result in an impaired function of CYP17A1 (17a-hydroxylase/17, 20-lyase), CYP19A1 (aromatase) and CYP1A2 *in vitro* [23,28–30]. Recently, a study on heme oxygenase I (HO-I) activity also indicated a decrease in CYPOR activity affected HO-I catalytic rate, and rate of bilirubin formation by HO-I enzyme is CYPOR-activity dependent [31]. Detailed saturated kinetic studies reveal that a decrease in HO-I activity is caused by a decrease in catalytic rate (*V*_max_), not substrate binding (*K*_m_) of HO-I [31]. Thus, we hypothesize the high catalytic activity of the mosquito CYP6AA3-mediated enzymatic activity found in the present study originates from a high electron transfer rate of rat CYPOR, C427R and L86F/L219F-flAnCYPOR activity compared to that of wildtype-flAnCYPOR enzyme. This, to our knowledge, is the first mosquito P450-mammalian CYPOR reconstitution that is more efficient than the native mosquito P450-CYPOR complex.

## 3. Experimental Section

### 3.1. Materials

Flavin mono-nucleotide (FMN), flavin-adenosine di-nucleotide (FAD), cytochrome *c*, nicotinamide adenosine diphosphate (NADP^+^), nicotinamide adenosine diphosphate reduced form (NADPH) and phenylmethylsulphonyl fluoride (PMSF) were purchased from Sigma-Aldrich (St. Louis, MO, USA). Isopropyl-β-d-thiogalactopyranoside (IPTG) was obtained from USB (Cleveland, OH, USA), Ni^2+^-NTA affinity column from Qiagen (Valencia, CA, USA), and Bio-Rad protein assay kit from Bio-Rad (Hercules, CA, USA). Quickchange site-directed Polymerase Chain Reaction (PCR) mutagenesis kit was purchased from Stratagene (LaJolla, CA, USA) and was used according to the manufacturer’s instructions.

### 3.2. Structure Prediction of Wild-Type and Mutant CYPOR

The amino acid sequence of wild-type *A. minimus* CYPOR was obtained from GenBank (ABL75156.1) and was used to search for template using PSI-BLAST against protein structures deposited in Brookhaven Protein Data Bank [32] for comparative modeling. Rat CYPOR-triple mutant (PDB:1JA1, [19]), exhibiting high resolved power of 1.80 Å and sharing 55% sequence identity to *A. minimus* CYPOR, was selected as the template. The first 63 residues were omitted from modeling since the *N*-terminus is associated with membrane anchor and membrane portion is absent in the template structure. Sequence alignment derived from ClustalW program of various CYPOR enzymes with some manual adjustment is shown in supplemental Figure 2. Alignments of AnCYPOR wild-type and mutants against the template were subsequently subjected to homology modeling using MODELLER9v6 [33]. A set of 1000 models for wild-type and mutant AnCYPORs was independently constructed. The cofactor FAD, FMN, and NADPH were positioned in target models as in the 1JA1 template. The most promising models of wild-type and mutant CYPORs were discriminated from incorrectly-folded structures using DOPE [34] and RAPDF [35] scoring functions. Candidate models were further energy minimized using AMBER ff03 all atom force field implemented in Amber10 [36] to remove bad van der Waals contacts. Model refinement was performed using steepest descent (SD) for 3000 iterations and followed by conjugated gradient (CG) minimizations until the energy gradient was less than 0.05 kcal/mol. The final energy minimized models were evaluated for conformation qualities using ProSAII [37,38] and Procheck [39]. Models were visualized and displayed using PyMOL (Schrödinger LLC, NY, USA).

### 3.3. Site-Directed Mutagenesis of AnCYPORs

Three amino acids were separately introduced into pET28a-L86F/L219FΔ55AnCYPOR plasmid DNA by Quickchange PCR mutagenesis kit as described in manufacturer’s instructions to generate triple mutants of the following substitutions: C427R, W678H and W678A. The sequences of primers used in this experiment are listed in Table 4. The same primer set was employed to generate C427R membrane-bound triple mutant using pTrc-L86F/L219F-flAnCYPOR plasmid DNA as template. All of the mutations were verified by DNA sequencing and the resultant plasmids were transformed into BL21 (DE3) *E. coli* cells.

### 3.4. Expression and Purification of AnCYPOR and Rat CYPOR Enzymes

Protein expression and purification of AnCYPORs were performed as previously described [14,15], while that of rat CYPOR followed Shen *et al*. (1989; [17]) with a slight modification. The *E. coli* C43 (DE3) carrying pIN-rat CYPOR plasmid was grown at 37 °C in TB broth containing 100 μg/mL ampicillin until OD^600^ was about 0.8 and protein expression induced by addition of 0.5 mM IPTG. Cells were allowed to grow for an additional 48 h and harvested by centrifugation at 5000× *g* for 15 min and resuspended in binding buffer (50 mM Tris pH 7.7, 0.1 M NaCl, 10% glycerol, and 20 mM imidazole) containing 0.2% Triton X-100. Cells were lysed by sonication and the supernatant was applied to a Ni^2+^-NTA affinity column previously equilibrated with the binding buffer. The column was extensively washed with binding buffer followed by the same buffer containing 30 mM imidazole. The protein was eluted by increasing imidazole concentration to 100 mM. Purity of protein was assessed by SDS-PAGE. Pure fractions were pooled, concentrated and stored at −80 °C until use. Protein concentration was determined by the Bio-Rad protein assay using bovine serum albumin (BSA) as standard.

### 3.5. Expression and Purification of CYP6AA3 from Insect-Baculovirus System

Recombinant baculovirus containing CYP6AA3 cDNA was used for protein expression in *Spodoptera frugiperda* (Sf9) insect cells as previously described [7]. Briefly, Sf9 cells were infected with the CYP6AA3 expressed virus (2.5 × 10^8^ plaque-forming units/mL) at the multiplicities of infection of 3. Infected cells were harvested at 70–80 h post infection and resuspended in sodium phosphate buffer pH 7.2 containing 1 mM EDTA, 0.5 mM PMSF, 5 μg/mL leupeptin, 0.1 mM DTT, and 20% glycerol, and subjected to microsome preparation using differential centrifugation. CYP6AA3 protein expression was observed by SDS-PAGE analysis. Total P450 content was measured from CO-different spectrum analysis [40].

### 3.6. Measurement of Flavin Contents and Activity Assay

Commercial FMN and FAD were further purified by high performance liquid chromatography (HPLC) and used for generation of a standard curve and for cofactor supplementation experiments. Concentrations of standard FAD and FMN solutions were determined spectrophotometrically at 450 nm using extinction coefficients of 11.3 and 12.2 mM^−1^ cm^−1^, respectively. FAD and FMN contents of each sample were measured using a fluorometric method as described previously [41]. The Bio-Rad protein assay was utilized to determine concentration of protein using rat CYPOR as standard. The rat CYPOR protein concentration was determined using spectrophotometric method [42]. The CYPOR-mediated cytochrome *c* reduction was carried out in 0.1 M Tris-HCl buffer, pH 7.5 as previously described [7] with minor modifications. After 1 min pre-incubation of enzyme in buffer with 40 μM cytochrome *c* at 25 °C, the reaction was initiated by addition of 50 μM NADPH. NADPH-dependent cytochrome *c* reduction was followed by a change in absorbance at 550 nm. Velocities are expressed as μmol/min/mg protein. One unit of enzyme was defined as the amount of enzyme catalyzing the reduction of 3 μmol of cytochrome *c* per minute under described conditions [43]. Substrate saturation steady-state kinetic studies of cytochrome *c* reduction were performed in 0.1 M Tris-Cl buffer, pH 7.5 at 25 °C in a final volume of 0.75 mL. Substrate saturation experiments were performed by varying NADPH concentration (2, 5, 7.5, 15, 25, 50 and 100 μM) and NADH concentration (0.3, 0.5, 1, 2, 5 and 10 mM). The reaction was started by addition of enzyme and the initial velocity data were analyzed by non-linear regression using the Grafit 6.0 software package.

### 3.7. CYP6AA3-Mediated BROD Assay

CYP6AA3-Mediated benzyloxyresorufin *O*-dealkylation reaction (BROD) was performed in 50 mM Tris-HCl buffer pH 7.5, in a total volume of 500 μL. Microsomes containing CYP6AA3 (~25 pmole) were used and enzymatic assays were reconstituted with either the purified AnCYPOR or rat CYPOR in the ratio of 3:1 and carried out with BR substrate (0.5, 1, 2, 4, 8 μM) as described [7]. Enzyme activity was measured with a RF-5301 PC spectrofluorophotometer (Shimadzu, Kyoto, Japan) at λ_ex_ = 530 and λ_em_ = 590 nm. The amount of resorufin product was calculated referring to the resorufin standard curve as described in Duangkaew *et al*., 2011 [9]. Apparent *K*_m_ and *V*_max_ values were estimated by non-linear regression analysis using GraphPad Prism 5 software package.

## 4. Conclusions

In summary, comparative modeling of AnCYPOR structure demonstrated differences in topology arrangement of AnCYPOR enzyme compared to rat CYPOR structure. AnCYPOR overall structure is comparable to rat CYPOR, however, numerous differences in amino acid residues and topology were found. Detail analysis revealed major differences in FMN- and FAD/NAD(P)H binding domains that might lead to differences in enzymatic properties and catalysis of mosquito CYPOR from mammalian CYPORs. Mutagenesis studies further indicated that C427 is critical for FAD binding in AnCYPOR. In addition, NAD(P)H binding and catalysis of this mosquito CYPOR is remarkably different from mammalian CYPORs. It is apparent that a low stoichiometry of FAD may not matter much in this enzyme, at least *in vitro*, as FAD can be easily reconstituted into the FAD binding site of AnCYPOR.

## Supplementary Information



## Figures and Tables

**Table 1:** Flavin content analysis of Δ55AnCYPOR.

Enzyme	FMN a	FAD a
Wild-type-Δ55AnCYPOR	0.52 ± 0.01	0.64 ± 0.03
L86F/L219F-Δ55AnCYPOR	0.97 ± 0.02	0.56 ± 0.01
L86F/L219F/C427R-Δ55AnCYPOR	0.98 ± 0.01	0.82 ± 0.02
L86F/L219F/W678A-Δ55AnCYPOR	0.96 ± 0.04	0.55 ± 0.02
L86F/L219F/W678H-Δ55AnCYPOR	0.97 ± 0.02	0.57 ± 0.04
Δ63AgCYPOR b	0.72 ± 0.01	0.80 ± 0.01
Human CYPOR b	0.88 ± 0.01	0.92 ± 0.02

a Flavin content is expressed as mean ± SD for mol of flavins per mol of protein in triplicate experiments. Standard flavin was measured and used for a plot of standard curve;

b Lian *et al.*, 2011 [18].

**Table 2:** Kinetic constants for cytochrome *c* reduction under substrate saturation condition by Δ55AnCYPOR enzymes.

Enzyme	Kinetic constant (μM) a		

	Cytochrome *c K*_m_	NADPH *K*_m_	NADH *K*_m_
Δ55AnCYPOR			
-wt	27.39 ± 1.41	9.61 ± 0.30	8.40 ± 0.10
-L86F/L219F	16.41 ± 2.22	6.49 ± 1.19	11.64 ± 1.44
-L86F/L219F/C427R	17.92 ± 1.79	7.02 ± 1.75	6.67 ± 2.23
-L86F/L219F/W678A	16.24 ± 1.56	3.34 ± 0.83	7.08 ± 2.06
-L86F/L219F/W678H	18.43 ± 2.12	1.91 ± 0.44	7.72 ± 1.33

a Values were obtained from substrate saturation steady-state kinetic studies in the presence of extra flavins as described in Materials and Methods and are means ± SD from triplicate experiments.

**Table 3:** The *in vitro* reconstitution assays of CYP6AA3 mosquito P450 with three different CYPOR enzymes.

CYPOR constructs	Cytochrome *c* reduction activity (U/mg protein) a	CYP3AA3-mediated BROD activity (pmole resorufin produced/min/pmol P450) a	
		*K*_m_ (μM)	*V*_max_ (min^−1^)
wt-AnCYPOR	0.35 ± 0.02	1.90 ± 0.61	3.10 ± 0.38
L86F/L219F-AnCYPOR	0.87 ± 0.04	1.89 ± 0.53	7.36 ± 0.78
L86F/L219F/C427R-AnCYPOR	1.62 ± 0.09	1.73 ± 0.17	10.20 ± 0.37
Rat CYPOR	18.55 ± 0.42	1.69 ± 0.24	17.40 ± 0.90

a Data are average of duplicate measurements.

**Table 4:** Primer used for mutagenesis study.

Constructs	Primer	Sequence
C427R	sense	5′-GGTACAAGACAGCCGCCGGAACGTAGTGCA-3′
	anti-sense	5′-TGCACTACGTTCCGGCGGCTGTCTTGTACC-3′
W678H	sense	5′-ACGTTACTCGGCGGACGTGCACAGCTAATCGACGGGCACA-3′
	anti-sense	5′-TGTGCCCGTCGATTAGCTGTGCACGTCCGCCGAGTAACGT-3′
W678A	sense	5′-ACGTTACTCGGCGGACGTGGCAAGCTAATCGACGGGCACA-3′
	anti-sense	5′-TGTGCCCGTCGATTAGCTTGCCACGTCCGCCGAGTAACGT-3′

Mutated codons are underlined.
