# Supplementary material for: Modeling of Anopheles minimus Mosquito NADPH-Cytochrome P450 Oxidoreductase (CYPOR) and Mutagenesis Analysis
Source: Int J Mol Sci. 2013 Jan 16;14(1):1788–801. doi: 10.3390/ijms14011788 (PMC3565348; doi:10.3390/ijms14011788)
Supplement: Supplementary file 1 [file ijms-14-01788-s001.pdf]

Supplementary Information

Figure S1. Ramachandran plot of a predicted AnCYPOR structure. The number of residues in the plot starts from Thr64 of the AnCYPOR amino acid sequence.

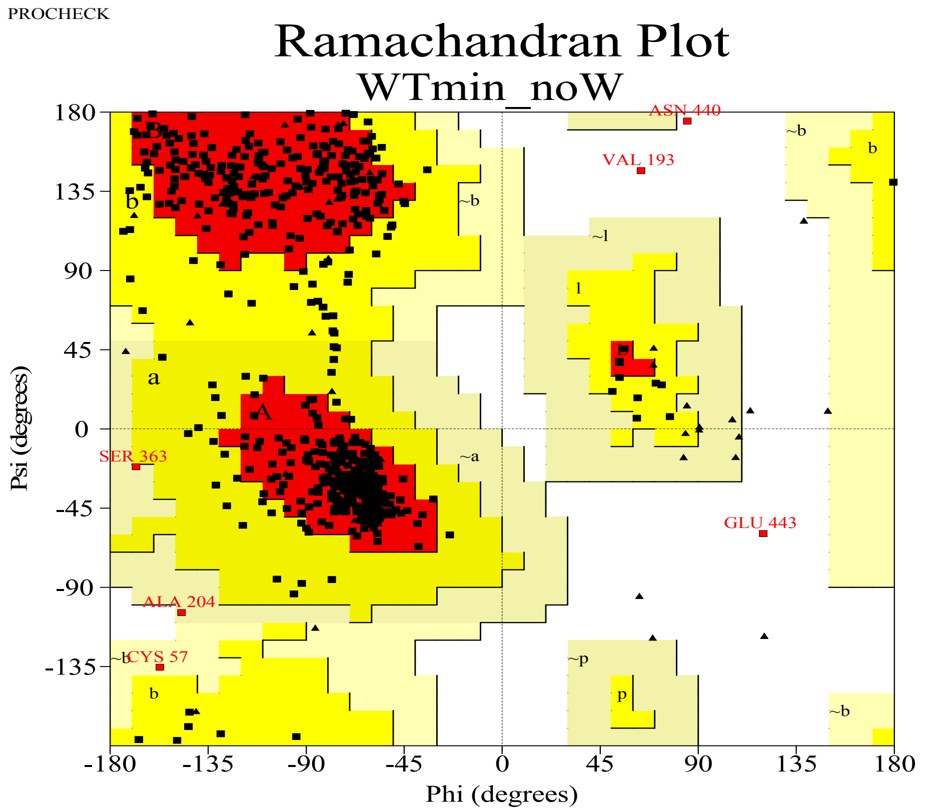

Plot statistics

Residues in most favoured regions [A,B,L]	458	84.3%
Residues in additional allowed regions [a,b,l,p]	79	14.5%
Residues in generously allowed regions [~a,~b,~l,~p]	3	0.6%
Residues in disallowed regions	3	0.6%
	----	------
Number of non-glycine and non-proline residues	543	100.0%
Number of end-residues (excl. Gly and Pro)	19	
Number of glycine residues (shown as triangles)	41	
Number of proline residues	30	

Total number of residues	633	

Based on an analysis of 118 structures of resolution of at least 2.0 Angstroms and R-factor no greater than 20%, a good quality model would be expected to have over 90% in the most favoured regions.

Figure S2. Sequence alignment of NADPH-cytochrome P450 oxidoreductases from various organisms. Amino acid sequences from CYPOR homologs of human (NCBI NP_000932.3), rat (NP_113764.1), fruit fly (*D. melanogaster*, NP_477158.1), and *An. gambiae* (AAO24765.1) were aligned with *An. minimus* CYPOR (ABL75156.1) and displayed by ClustalW. The residue numbers of the sequences are shown. Identical amino acids are represented as dashes. The regions previously reported as the binding sites of the coenzymes are boxed.

```
An. minimus          MDAQAETEMPTGSVS   DEPFLGPLDIILLVCLLAGTAWYLLKGKKKENQASQFKS 54
An. gambiae          ----T---V-A----   ----------V---S------W---------S------- 54
D. melanogaster      -ASEQTIDGAAAIP-GGG------L--VA—AV—IG--A-F-F-RSR---EEP  TR- 55
H. sapiens        MIN-GDSHVDTSS-V-EAVAEEVSLFSMT-M—FSLIVGLLTYWF--FR----EVP      55
R. norvegicus        -GDSH-DTSA-MPEAVAEEVSLFSTT-MV-FSLIVGVLTYWFIFRK---EIP      52
```

Figure S2. *Cont.*

```
An. minimus          YSIQPTTVNTMTMVENSFIKKLQSSGRRLVVLYGSQTGTAEEFAGRLAKEGIRYQMKGMV 114
An. gambiae          --------------------------------F--------------------------- 114
D. melanogaster      --------C-TSASD-------KA---S---F-------G-------------RL---- 115
H. sapiens            EFTKIQTL-SSVR-S--VE-MKKT-NII--F------------N--S-DAH--GMR--S 114
R. norvegicus         EFSKIQTTAPPVK-S--VE-MKKT--NII-F------------N--S-DAH--GMR--S 111
                                                        FMN

An. minimus          ADPEECNMEELLMLKDIDKSLAVFCLATYGEGDPTDNCMEFYDWIQNNDLDMTGLNYAVF 174
An. gambiae          ------------------------------------------------------------ 174
D. melanogaster      ------D-----Q-----N-----------------A----E--TSG-V-LS------- 175
H. sapiens           -----YDLAD-SS-PE--NA-V---M-----------AQD----L-ET-V-LS-VKF--- 174
R. norvegicus        -----YDLAD-SS-PE-----V---M-----------AQD----L-ET-V-L--VKF--- 171
                                                            FMN

An. minimus          GLGNKTYEHYNKVGIYVDKRLEELGANRVFELGLGDDDANIEDYLITWKEKFWPTVCDFF 234
An. gambiae          ---------------------------------------------F-------------Y- 234
D. melanogaster      -------------A------------------------------DF----DR---A---H- 235
H. sapiens           ---------F-AM-K-------Q---Q-I---------G-L-EDF---R-Q---A--EH- 234
R. norvegicus        ---------F-AM-K---Q---Q---Q-I---------G-L-EDF---R-Q---A--EF- 231
                             FMN

An. minimus          GIESTGEDVLMRQYRLLEQPEVGADRIYTGEVARLHSLQTQRPPFDAKNPFLAPIKVNRE 294
An. gambiae          --------------------D-S------------------------------------ 294
D. melanogaster      ---GG--E-I----------D-QP-------I-----I-N------------------- 295
H. sapiens           -V-A---ESSIR--ELVVHTDID-AKV-M--MG--K-YEN-K-----------AVTT--K 294
R. norvegicus        -V-A---ESSIR--ELVVHEDMDVAKV-T--MG--K-YEN-K-----------AVTA--K 291

An. minimus          LHKAGGRSCMHVEFDIEGSKMRYEAGDHLAMYPVNDRDLVERLGKLCNADLETVFSLINT 354
An. gambiae          --------------------------------------------R----E-D-------- 354
D. melanogaster      ---G-------I-LS--------D----V--F----KS---K--Q------D-------- 355
H. sapiens           -NQGTE-HL--L-L--SD--I--ES---V-V--A--SA--NQ--KILG---DV-M--N-L 354
R. norvegicus        -NQGTE-HL--L-L--SD--I--ES---V-V--A--SA--NQI-EILG---DVIM--N-L 351

An. minimus          DTDSSKKHPFPCPTTYRTALTHYLEITALPRTHILKELAEYCSEEKDKEFLRFISSTAPE 414
An. gambiae          -----------------------------------------G----------------D 414
D. melanogaster      ----------------------------I-------------TD-E---L--SMA-IS-- 415
H. sapiens           -EE-N---------S------Y--D--NP---NV-Y---Q-A--PSEQ-L--KMA-SSG- 414
R. norvegicus        -EE-N----------------Y--D--NP---NV-Y---Q-A--PSEQ-H-HKMA-SSG- 411

An. minimus          GKAKYQEWVQDSCRNVVHVLEDIPSCHPPIDHVCELLPRLQPRYSSISSSSKIHPTTVHV 474
An. gambiae          ---------------I-----------------------------H-------L------ 474
D. melanogaster      --E---S-I--A---I--I----K--R-------------------Y-----A-L---D--- 475
H. sapiens           --EL-LS--VEAR-HILAI-Q-CP-LR-----L---------A--Y--A----V--NS--I 474
R. norvegicus        --EL-LS--VEAR-HILAI-Q-YP-LR-----L---------A--Y--A----V--NS--I 471
                                                                         FAD
```

Figure S2. *Cont.*

```
An. minimus          TAVLVKYETKTGRLNKGVATTFLAEKHPNDGEP LPRVPIFIRKSQFRLPPKPETPVIMV 533
An. gambiae          --------------------------------- A------------------------ 533
D. melanogaster      -----E-K-P---I-------Y-KN-Q-QGS—EVK----V----------T-------- 533
H. sapiens           C--V-E----A--I------NW-RA-E-AGENGGRAL--M-V--------F-AT------ 534
R. norvegicus        C--A-E--A-S--V------SW-RA-E-AGENGGRAL--M-V--------F-ST------ 531
                                                                            NADPH

An. minimus          GPGTGLAPFRGFIQERDFSKQEGKDIGQTTLYFGCRKRSEDYIYEDELEDYSKRGIIN L 592
An. gambiae          ----------------HC-----E--------------------------------- - 592
D. melanogaster      ----------------QFLRD---TV-ESI---------------S---EWV-K-TL- - 592
H. sapiens           -----V---I------AWLRQQ--EV-E-L--Y---RSD---L-RE--AQFHRD-ALTQ- 594
R. norvegicus        -----I---M------AWLREQ--EV-E-L--Y---RSD---L-RE--ARFH-D-ALTQ- 591
                          NADPH

An. minimus          RVAFSRDQDKKVYVTHLLEQDSDLIWNVIGENKGHFYVCGDAKNMATDVRNILLKVIRSK 652
An. gambiae          --------E----------------S----------I----------------------- 652
D. melanogaster      KA------G-----Q------A---------------I--------V-------V-I-ST- 652
H. sapiens           N-----E-SH----Q---K--REHL-KL- -GGA-I------R---R--Q-TFYDIVAEL 653
R. norvegicus        N-----E-AH----Q---KR-REHL-KL-H-GGA-I------R---K--Q-TFYDIVAEF 651
                                                NADPH

An. minimus          GGLSETEAQQYIKKMEAQKRYSADVWS 679
An. gambiae          --------------------------- 679
D. melanogaster      -NM--AD-V------------------ 679
H. sapiens           -AMEHAQ-VD----LMTKG---L---- 680
R. norvegicus        -PMEH-Q-VD-V--LMTKG---L---- 678
```

© 2013 by the authors; licensee MDPI, Basel, Switzerland. This article is an open access article distributed under the terms and conditions of the Creative Commons Attribution license (http://creativecommons.org/licenses/by/3.0/).
